# Evaluation of 3β-hydroxysteroid dehydrogenase activity using progesterone and androgen receptors-mediated transactivation

**DOI:** 10.3389/fendo.2024.1480722

**Published:** 2024-10-02

**Authors:** Takashi Yazawa, Yugo Watanabe, Yuko Yokohama, Yoshitaka Imamichi, Kazuya Hasegawa, Ke-ichi Nakajima, Takeshi Kitano, Takanori Ida, Takahiro Sato, Mohammad Sayful Islam, Akihiro Umezawa, Satoru Takahashi, Yasuhito Kato, Sharmin Jahan, Jun-ichi Kawabe

**Affiliations:** ^1^ Department of Biochemistry, Asahikawa Medical University, Asahikawa, Japan; ^2^ Department of Obstetrics and Gynecology, Asahikawa Medical University, Asahikawa, Japan; ^3^ Department of Marine Science and Technology, Fukui Prefectural University, Obama, Japan; ^4^ Faculty of Health and Medical Science, Teikyo Heisei University, Tokyo, Japan; ^5^ Department of Biological Sciences, Graduate School of Science and Technology, Kumamoto University, Kumamoto, Japan; ^6^ Division of International Cooperation and Education, Center for Animal Disease Control, University of Miyazaki, Miyazaki, Japan; ^7^ Division of Molecular Genetics, Institute of Life Sciences, Kurume University, Kurume, Japan; ^8^ Department of Pharmacy, Mawlana Bhashani Science and Technology University, Tangail, Bangladesh; ^9^ Department of Reproduction, National Research Institute for Child Health and Development, Tokyo, Japan; ^10^ Department of Pediatrics, Asahikawa Medical University, Asahikawa, Japan; ^11^ Department of Endocrinology, Bangabandhu Sheikh Mujib Medical University (BSMMU), Dhaka, Bangladesh

**Keywords:** HSD3B2, CAH, progesterone, androstenedione, DSD

## Abstract

3β-Hydroxysteroid dehydrogenases (3β-HSDs) catalyze the oxidative conversion of delta (5)-ene-3-beta-hydroxy steroids and ketosteroids. Human 3β-HSD type 2 (HSD3B2) is predominantly expressed in gonadal and adrenal steroidogenic cells for producing all classes of active steroid hormones. Mutations in HSD3B2 gene cause a rare form of congenital adrenal hyperplasia with varying degree of salt wasting and incomplete masculinization, resulting from reduced production of corticoids and androgens. Therefore, evaluation of the HSD3B2 enzymatic activity in both pathways for each steroid hormone production is important for accurately understanding and diagnosing this disorder. Using progesterone receptor (PR)- and androgen receptor (AR)-mediated transactivation, we adapted a method that easily evaluates enzymatic activity of HSD3B2 by quantifying the conversion from substrates [pregnenolone (P5) and dehydroepiandrosterone (DHEA)] to (progesterone and androstenedione). HEK293 cells were transduced to express human HSD3B2, and incubated medium containing P5 or DHEA. Depending on the incubation time with HSD3B2-expressing cells, the culture media progressively increased luciferase activities in CV-1 cells, transfected with the PR/AR expression vector and progesterone-/androgen-responsive reporter. Culture media from human and other mammalian HSD3B1-expressing cells also increased the luciferase activities. HEK293 cells expressing various missense mutations in the HSD3B2 gene revealed the potential of this system to evaluate the relationship between the enzymatic activities of mutant proteins and patient phenotype.

## Introduction

Steroid hormones are produced from cholesterol via serial enzymatic reactions catalyzed by cytochrome P450 hydroxylases (CYPs) and hydroxysteroid dehydrogenases (HSDs) (1, 2). Among these enzymes, 3β-HSDs belong to the short*-*chain dehydrogenase/reductase superfamily of proteins, which requires NAD(P)+ as a co-factor in the reactions (3). With CYP11A1, a rate-limiting enzyme of steroidogenesis, they are essential for producing all classes of steroid hormones by catalyzing 3β-hydroxysteroid dehydrogenation and Δ^5^- to Δ^4^-isomerisation of the Δ^5^-steroid precursors. 3β-HSDs synthesize an active steroid and its precursors by converting pregnenolone (P5), 17α-pregnenolone (17-OHP5) and dehydroepiandrosterone (DHEA) into progesterone (P4), 17α-progesterone (17-OHP4) and androstenedione (A4), respectively. Although P4 itself is an active steroid hormone that strongly activates progesterone receptor (PR), it also acts as a precursor of mineralocorticoid (aldosterone) for regulating blood pressure through sodium reabsorption. 17-OHP4 and A4 are the precursors for glucocorticoid (cortisol) and androgen (testosterone) (4), respectively.

Multiple types of HSD3B/Hsd3b exist in species-specific manner (3). In human, there are two isozymes, HSD3B1 and HSD3B2 (5). Human HSD3B1 is mainly expressed in placenta and involved in the production of progesterone for maintaining the pregnancy (3, 6). It is also expressed at minor extent in other tissues, such as skin, breast and prostate. Although some single nucleotide polymorphisms (SNPs) are likely involved in prostate and breast cancers (7–13), deficiency of HSD3B1 has not been reported. In contrast, HSD3B2 is almost exclusively expressed in primary steroidogenic tissues, such as adrenal gland and gonads (testis and ovary) (3, 14). Deficiency caused by the mutations in HSD3B2 gene results in congenital adrenal hyperplasia (CAH) with salt-wasting (SW) and ambiguous genitalia, due to the reduction of glucocorticoid, mineralocorticoid and androgen (15). Although HSD3B2 deficiency is a rare autosomal recessive disorder, the clinical presentation of SW and genitalia is heterogenous among different mutations (3, 15, 16). In particular, SW phenotype ranges from no symptoms to severe forms. Therefore, the evaluation of HSD3B2 enzymatic activities is important for understanding and diagnosing this disorder. However, as mentioned above, because HSD3B2 catalyzes multiple steps in the production of various steroid hormones, evaluation of the enzymatic activities using the traditional methods like radioactive isotopes and liquid chromatography-mass spectrometry-mass spectrometry (LC–MS/MS) is complicated. We recently reported a method that easily evaluates the activities of testosterone-producing enzyme HSD17B3 using androgen receptor (AR)-mediated transactivation in cultured cells (17). Based on this methodology, we established a method in this study to evaluate HSD3B2 activities in converting substrates (P5 and DHEA) into the precursors (P4 and A4) of important steroid hormones (aldosterone and testosterone) relevant to each clinical symptom. Using this system, we evaluated the enzymatic activities of various missense mutants of HSD3B2 proteins-derived from the patients with or without SW symptom.

## Materials and methods

### Cell culture, transfection and luciferase assay

CV-1 and HEK293 cells were cultured in DMEM supplemented with 10% fetal bovine serum (FBS). Cells were transfected using HilyMax (Dojindo Laboratories, Kumamoto, Japan). One day before transfection, cells were seeded on 48-well plates and cultured with DMEM supplemented with 10% Hyclone Charcoal/Dextran treated FBS (GE Healthcare UK Ltd, Buckinghamshire, England). At 24 h post-transfection, the cells were treated with vehicle (EtOH), each steroid, or supernatant of culture media for 24 h. Luciferase assays were performed as described previously (18, 19). Each data point represents the mean of at least four independent experiments.

### Western blotting analysis

Extraction of total proteins from cultured cells and subsequent quantification were conducted as described previously (20, 21). Equal amounts of protein (30 μg) were resolved using 12.5% SDS-PAGE and transferred to polyvinylidene difluoride membranes. Western blot analyses of HSD3B2, FLAG and GAPDH were performed using antibodies directed against HSD3B2 (67572-1-Ig, Proteintech Group, Inc., Rosemont, IL, USA), FLAG (Clone 871701, R&D Systems, Inc., Minneapolis, MN, USA) and GAPDH (14C10; Cell Signaling Technology, Inc.), respectively. Clarity Western ECL Substrate (Bio-Rad Laboratories Inc., Hercules, CA, USA) were used for detection.

### Plasmids

The pCMV-2B expressing human HSD3B1 and HSD3B2 were generated by cloning the open reading frame (ORF) of each gene into a pCMV-2B vector (Invitrogen, Carlsbad, CA, USA). The pQCXIP expressing ovine HSD3B1 and guinea pig Hsd3b1 were generated by cloning the ORF of each gene into a pQCXIP vector. Constructs having mutations in HSD3B2 gene were prepared by the QuikChange Site-Directed Mutagenesis Kit (Stratagene, La Jolla, CA, USA) with each primer (**Supplementary Table 1**). The pcDNA3 expressing human PR was generated by cloning the ORF of human PR into a pcDNA3 vector. A Slp-ARU/Luc reporter and pQCXIP/human AR were prepared as described (22).

### Measurements by liquid chromatography-tandem spectrometry

P5, P4, DHEA and A4 in culture media were quantified using LC-MS/MS are based on methods as we previously described (23). As internal standards, P5, P4, DHEA and A4 were added to a medium diluted with distilled water. The steroids were extracted with methyl *tert*-butyl ether (MTBE). After evaporating the MTBE layer to dryness, the extract was dissolved in 0.5 mL of methanol and then diluted with 1 ml of distilled water. The sample was applied to OASIS MAX cartridge which had been successively conditioned with 3 ml of methanol and 3 ml of distilled water. After the cartridge was washed with 1 ml of distilled water, 1 ml of methanol/distilled water/acetic acid (45:55:1,v/v/v), and 1 ml of 1% pyridine solution, the steroids were eluted with 1 ml of methanol/pyridine (100:1,v/v). After evaporation, the residue was reacted with 50 μl of mixed solution (80 mg of 2-methyl-6-nitrobenzoic anhydride, 20 mg of 4-dimethylaminopyridine, 40 mg of picolinic acid and 10 μl of triethylamine in 1 ml of acetonitrile) for 30 min at room temperature. After the reaction, the sample was dissolved in 0.5 ml of ethyl acetate/hexane/acetic acid (15:35:1, v/v) and the mixture was applied to HyperSep Silica cartridge which had been successively conditioned with 3 mL of acetone and 3 ml of hexane. The cartridge was washed with 1 mL of hexane, and 2 mL of ethyl acetate/hexane (3:7, v/v). P5, P4, DHEA and A4were eluted with 2.5 ml of acetone/hexane (7:3, v/v). After evaporation, the residue was dissolved in 0.1 ml of acetonitrile/distilled water (2:3, v/v) and the solution was subjected to a LC-MS/MS.

### Statistical analyses

Data are presented as the mean ± SEM or the mean ± SD. Differences between groups were assessed by the Student’s t-test or the one-way ANOVA followed by Tukey’s multiple comparison test using EZR (Easy R, Saitama Medical Center, Jichi Medical University, Saitama, Japan), a graphical user interface for R (The R Foundation for Statistical Computing, Vienna, Austria) (24). p-Values less than 0.05 were considered significant.

## Results

### Establishment of a method to evaluate enzymatic activities of HSD3B2 using cell-based assays

**Figure 1:**
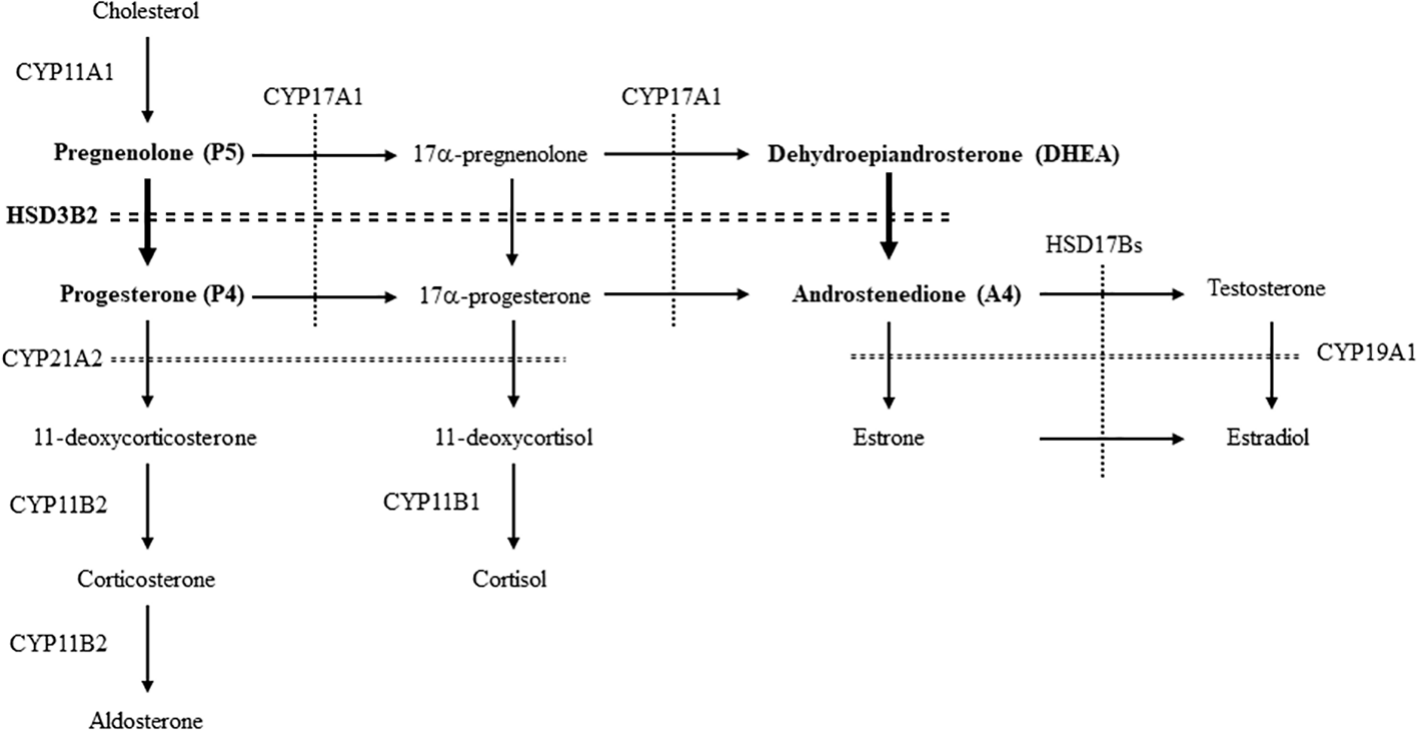
The enzymes and pathways for the synthesis of steroid hormones from cholesterol in human. The pathways involved in this study are showed by bold letters and arrows.

**Figure 2:**
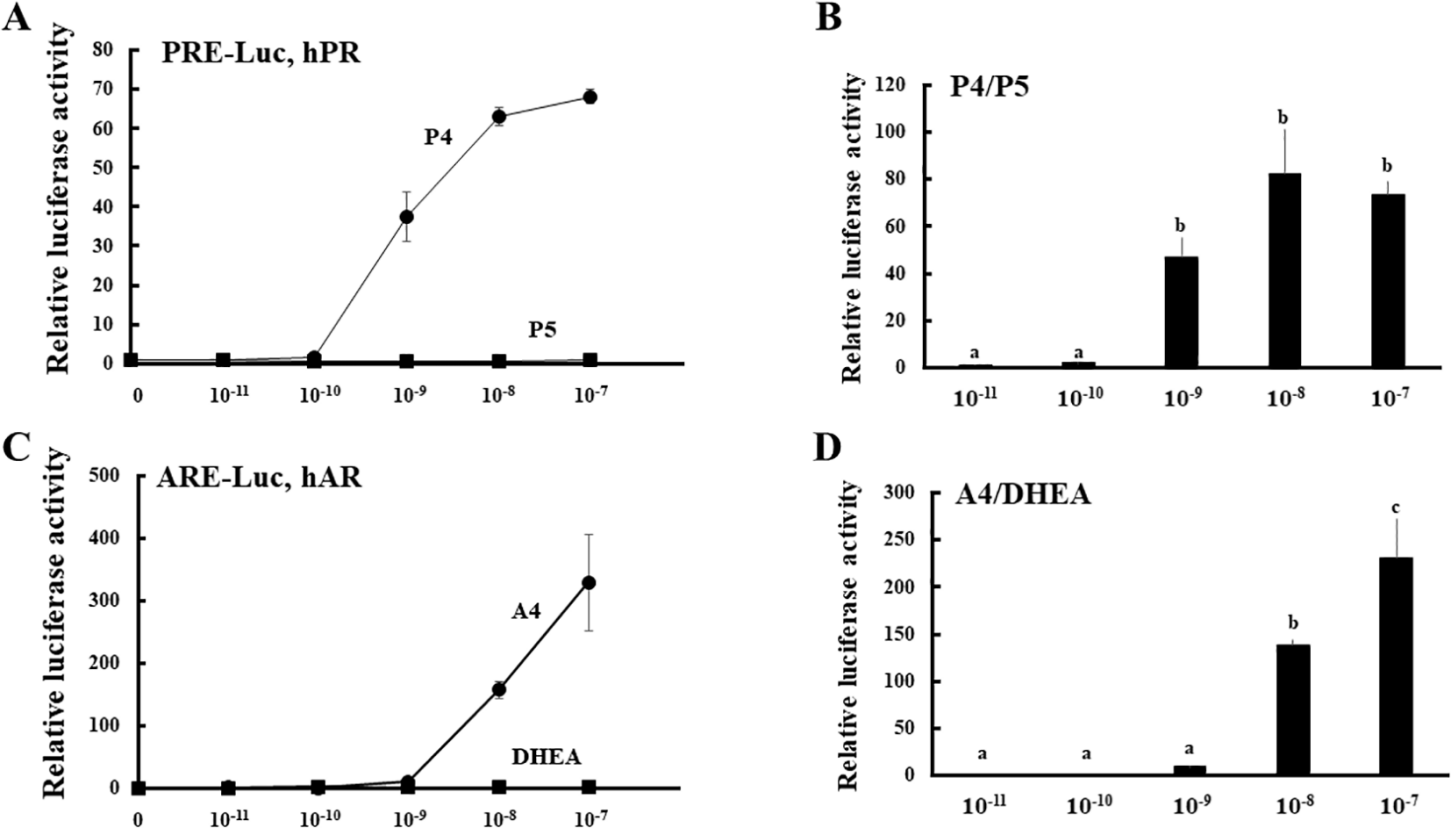
PR- and AR-mediated transactivation by substrates (P5 and DHEA) and products (P4 and A4) of HSD3B2 in CV-1 cells. **(A)** CV-1 cells were transfected with the PRE-Luc vector and the human PR-expression vector. At 24 h post-transfection, cells were incubated with or without increasing concentrations of each steroid for 24 h. Data represent the mean ± sem of at least four independent experiments. **(B)** Comparison for the potentials of PR-mediated transactivation between P5 and P4 at each concentration. Values of P5 were defined as 1. Values marked by the different letters (a, b, c) are significantly different with each other (*P* < 0.05). **(C)** CV-1 cells were transfected with the ARE-Luc vector and the human AR-expression vector. At 24 h post-transfection, cells were incubated with or without increasing concentrations of each steroid for 24 h. Data represent the mean ± sem of at least three independent experiments. **(D)** Comparison for the potentials of AR-mediated transactivation between DHEA and A4 at each concentration. Values of DHEA were defined as 1. Values marked by the different letters (a, b, c) are significantly different with each other (*P* < 0.05).

To establish a novel system for evaluating the enzymatic activities of HSD3B2 in multiple pathways (**Figure 1**), we compared the potential for PR- and AR-mediated transactivation between substrates (P5 and DHEA) and products (P4 and A4) at various concentrations in CV-1 cells (**Figure 2**). PR-mediated transactivation was markedly increased from 10^-9^ M by P4, whereas the transactivation was not increased by P5 at any concentration (**Figure 2A**). The ratio of P4 to P5-induced activity was also elevated from 10^-9^ M and the peak value was observed at 10^-8^ M. DHEA almost completely unaffected the AR-mediated transactivation in all the examined concentrations (**Figure 2B**). In contrast, A4 increased it from 10^-8^ M. Consistent with this, the ratio of A4 to DHEA-induced activity was significantly elevated at 10^-8^ and 10^-7^ M (**Figure 2B**). Based on these results, we selected 10^-8^ M in following experiments as optimal concentration to discriminate between substrates and products in the PR- or AR-mediated transactivation system.

**Figure 3:**
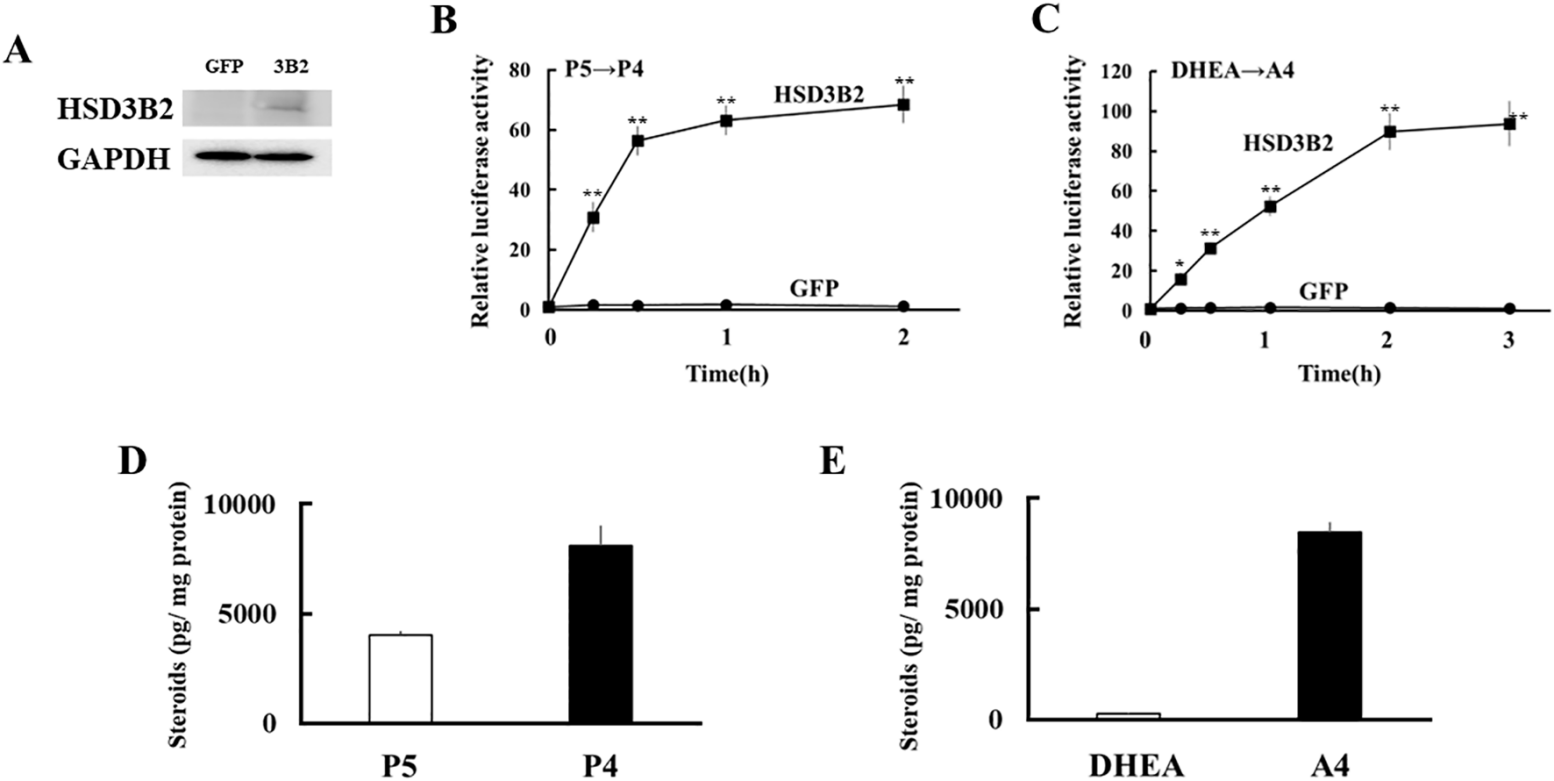
Development of a novel method to detect enzymatic activity of HSD3B2 by cell-based assay. **(A)** Western blot analyses were performed with the antibodies against HSD3B2 and GAPDH using lysates derived from GFP- or HSD3B2-expressed HEK293 cells. **(B)** Activation of human PR-mediated transcription by culture media from GFP- or HSD3B2-expressed HEK293 cells. CV-1 cells were transfected with PRE-Luc and human PR-expression vectors. At 24 h post-transfection, cells were incubated with each culture medium collected at indicated time after P5 (10 nM) addition for 24 h. Values for P5 (10 nM) addition in PRE-Luc vector-and human PR-expression vector-transfected CV-1 cells were defined as 1. Data represent the mean ± sem of at least four independent experiments. Differences between GFP and HSD3B2 groups in each time point are indicated by **p < 0.01. **(C)** Activation of human AR-mediated transcription by culture media from GFP- or HSD3B2-expressing HEK293 cells. CV-1 cells were transfected with ARE-Luc and human AR-expression vectors. At 24 h post-transfection, cells were incubated with each culture medium collected at indicated time after DHEA (10 nM) addition for 24 h. Values for DHEA (10 nM) addition in ARE-Luc vector-and human AR-expression vector-transfected CV-1 cells were defined as 1. Data represent the mean ± sem of at least four independent experiments. Differences between GFP and HSD3B2 groups in each time point are indicated by *p < 0.05 and **p < 0.01. **(D, E)** Concentrations of P5/P4 **(D)** and DHEA/A4 **(E)** in culture medium from HSD3B2-expressed HEK293 cells at 2 h and 3 h after addition of P5 (10 nM) and DHEA (10 nM), respectively. Each column represents the mean ± SEM of three independent experiments.

**Figure 4:**
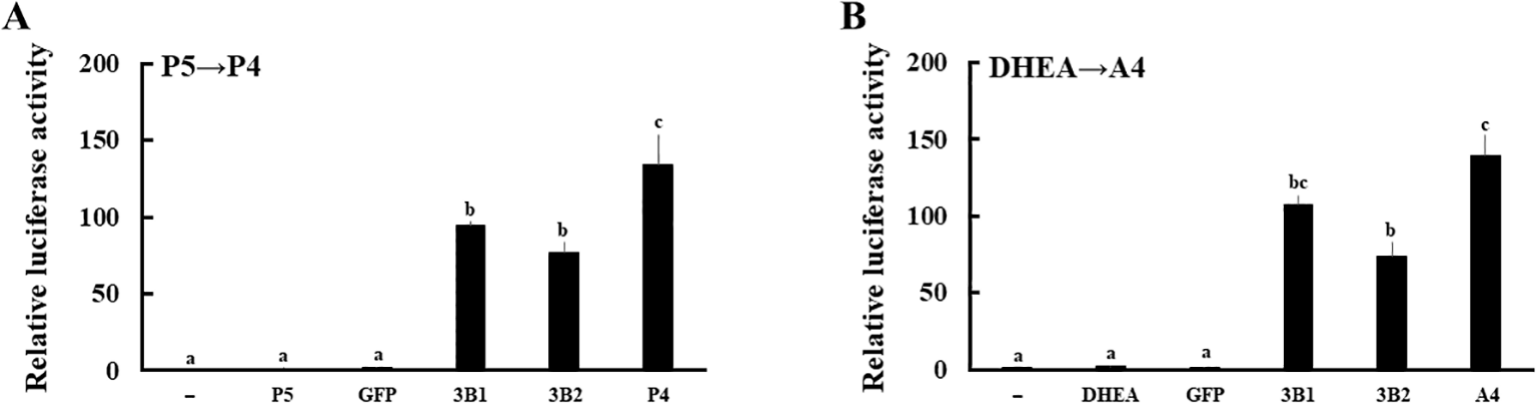
Evaluation of the enzymatic activities of human HSD3B1 and HSD3B2 using culture media from each gene-transfected HEK293 cells. Activation of human PR- and AR-mediated transcription by HSD3B1 and HSD3B2 using culture media from each gene-transfected HEK293 cells. CV-1 cells were transfected with PRE-Luc/human PR-expression vectors **(A)** and ARE-Luc/human AR-expression vectors **(B)**. At 24 h post-transfection, cells were incubated for 24 h with vehicle (lane -), P5 (10 nM), P4 (10 nM), DHEA (10 nM),A4 (10 nM), culture medium from GFP or each HSD3B-expressing HEK293 cells collected at 2 h and 3 h after addition of P5 (10 nM) and DHEA (10 nM), respectively. Values of the vehicle were defined as 1. Data represent the mean ± sem of at least four independent experiments. Values marked by the different letters (a, b, c) are significantly different with each other (*P* < 0.05).

Next, expression vectors of GFP and human HSD3B2 genes were transfected into HEK293 cells (**Figure 3A**). Two days post-transfection, P5 or DHEA was added to the culture medium at 10^-8^ M for 3h. Then, the supernatants were collected at each time point, and transfer to culture plates of CV-1 cells that were transfected with PRE reporter/human PR expression vector or ARE reporter/human AR expression vector. Each culture medium-derived from HSD3B2-transfected HEK293 cells increased PR- and AR-mediated transactivation in CV-1 cells in a time-dependent manner after the substrate addition (**Figures 3B, C**). PR-mediated transactivation in CV-1 cells reached plateau in the medium cultured for 30 min with P5, whereas AR-mediated transactivation in CV-1 cells was reached plateau in the medium cultured for 2h with DHEA. Consistent with these results, P5 and DHEA were markedly converted to P4 (**Figure 3D**) and A4 (**Figure 3E**), respectively, at the end of incubation, despite these products were undetectable at the beginning of incubation (data not shown). In contrast, culture media-derived from GFP-transfected HEK293 cells did not induce the luciferase activities at all time points. These results indicate that HSD3B2-mediated conversion of P5 into P4, and DHEA into A4, in culture media are able to evaluate by PR- and AR-mediated transactivation in this system. It can also evaluate the activities of human HSD3B1 that was transiently expressed in HEK293 cells (**Figure 4**; **Supplementary Figure 1**). Activities of human HSD3B1were higher than the activities of HSD3B2, despite statistically not significant. In addition to human proteins, the activities of ovine HSD3B1 and guinea pig Hsd3b1 were detectable using this system (**Supplementary Figures 2A, B**).

### Enzymatic activities of various missense mutations in the HSD3B2 gene

**Figure 5:**
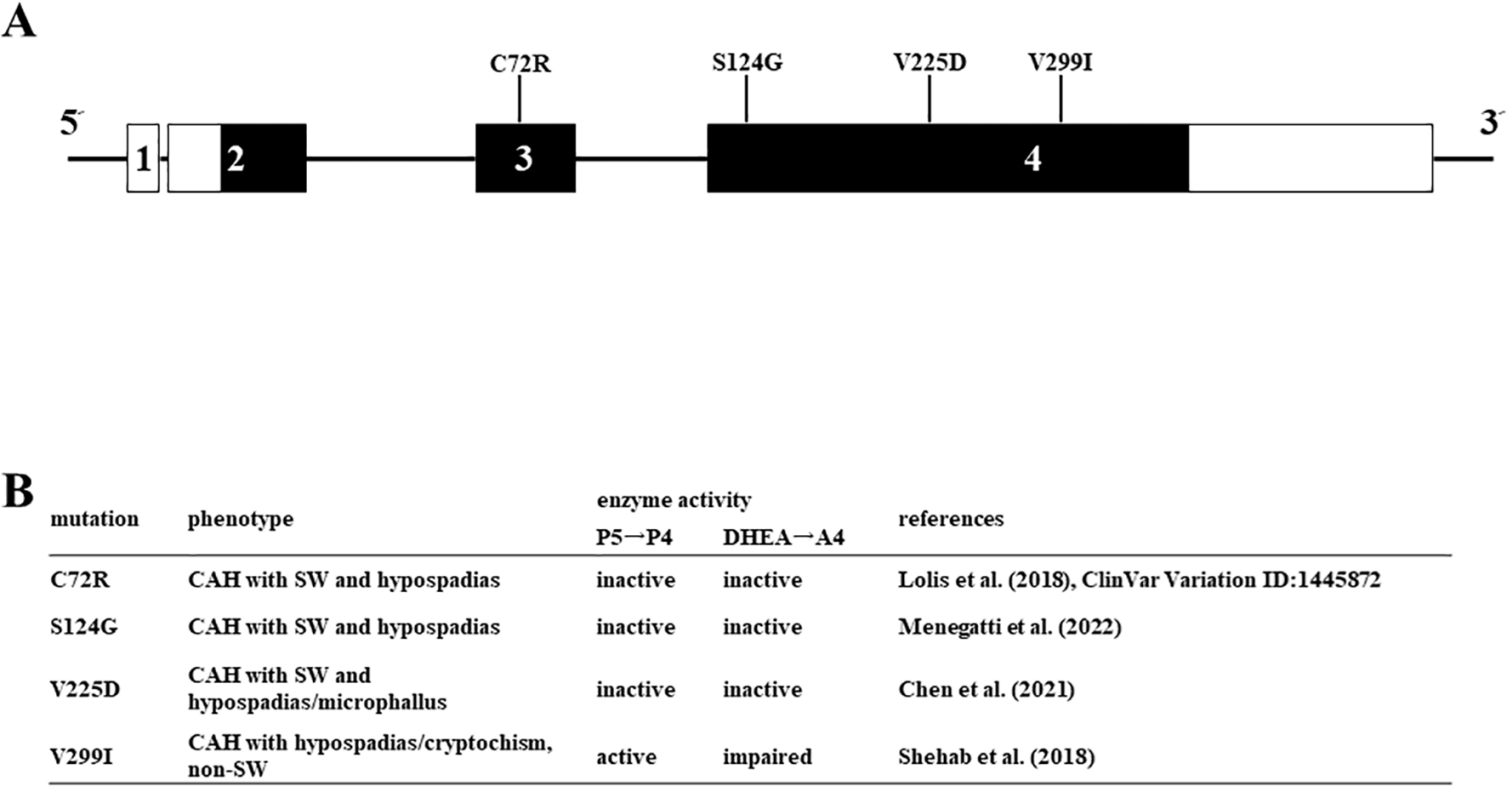
Missense mutations in HSD3B2 genes investigated in this study. **(A)** Schematic structure of the human HSD3B2 gene and substitution of amino acids. The numbered box indicates exons 5’ and 3’-untranslated regions are indicated by white bars. **(B)** Clinical symptoms and enzymatic activities of each mutation in the HSD3B2 gene.

**Figure 6:**
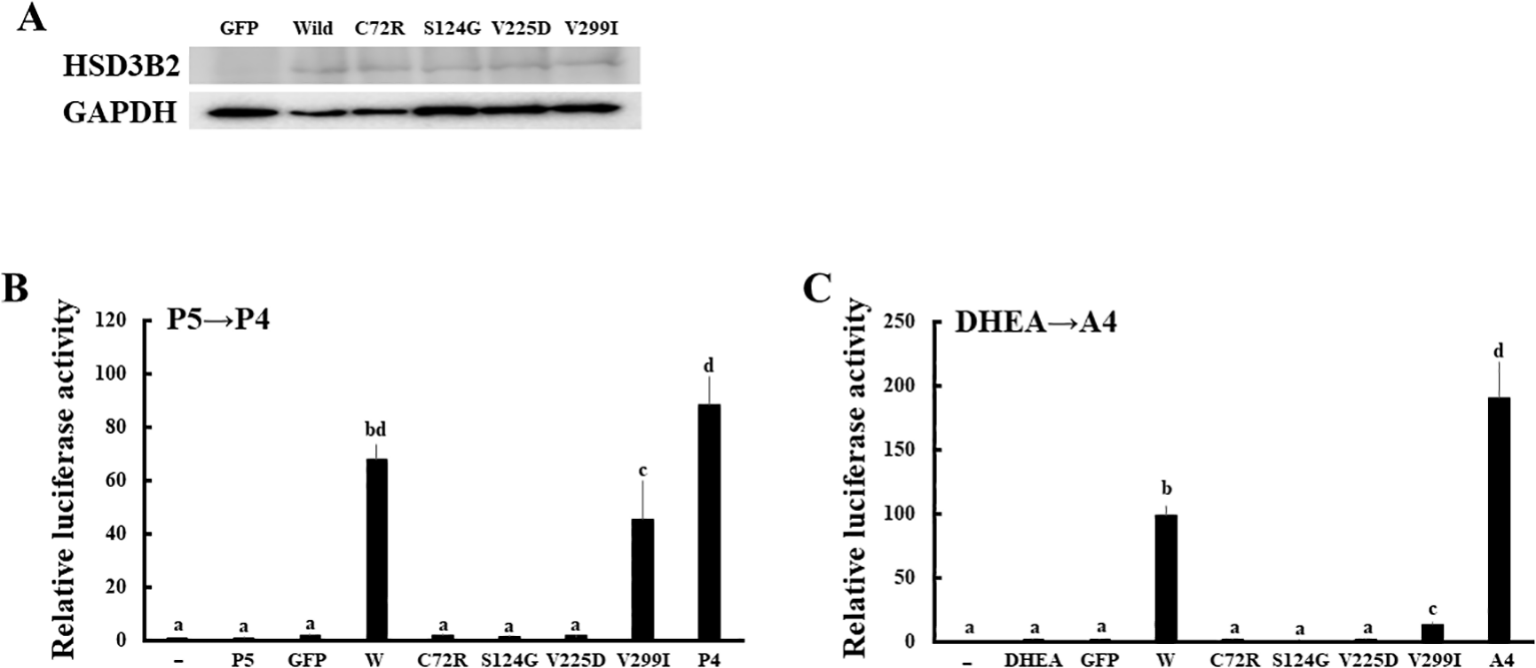
Enzymatic activities of missense mutations in HSD3B2 gene. **(A)** Expression of wild-type and mutant HSD3B2 enzymes. Western blot analyses were performed with the antibodies against HSD3B2 and GAPDH using lysates of GFP-, wild-type HSD3B2- and each mutant HSD3B2-expressed HEK293 cells. **(B)** Activation of human AR-mediated transcription by culture media from wild-type and mutant HSD3B2-expressing HEK293 cells. CV-1 cells were transfected with PRE-Luc and human PR-expression vectors. At 24 h post-transfection, cells were incubated with each culture medium collected at 2 h after P5 (10 nM) addition for 24 h. Values for vehicle addition in PRE-Luc vector-and human PR-expression vector-transfected CV-1 cells were defined as 1. Data represent the mean ± sem of at least four independent experiments. Values marked by the different letters (a, b, c, d) are significantly different with each other (*P* < 0.05). **(C)** Activation of human AR-mediated transcription by culture media from wild-type and mutant HSD3B2-expressing HEK293 cells. CV-1 cells were transfected with ARE-Luc and human AR-expression vectors. At 24 h post-transfection, cells were incubated with each culture medium collected at 3 h after DHEA (10 nM) addition for 24 h. Values for vehicle addition in ARE-Luc vector-and human AR-expression vector-transfected CV-1 cells were defined as 1. Data represent the mean ± sem of at least four independent experiments. Values marked by the different letters (a, b, c) are significantly different with each other (*P* < 0.05). Values marked by the different letters (a, b, c, d) are significantly different with each other (*P* < 0.05).

We applied the above system to evaluate the enzymatic activities of four HSD3B2 mutants with or without SW (**Figure 5**). Although these missense mutants are identified within a decade (25–28), the enzymatic activities of mutant proteins had not been defined. Therefore, we transfected these amino acid substituted enzymes in HEK293 cells, and compared the enzymatic activities with wild type protein using the above system (**Figure 6A**). Consistent with patient symptoms (genitalia dysgenesis), AR-mediated transactivation by culture media adding DHEA in each mutant gene-transfected cells were markedly decreased compared with those in wild type protein transfected cells (**Figure 6C**). C72R, S124G and M225V completely disappeared the enzymatic activities, whereas V299I had some residual activities (19.9% versus wild type). In contrast to AR-mediated transactivation, PR-mediated transactivation by culture media in each mutant gene-transfected cells showed some difference between mutants (**Figure 6B**). It was never induced by culture media from C72R-, S124G- and M225V-transfected cells, whereas culture media from V299I transfected cells induced the luciferase activities despite some lesser than those from wild type gene transfected cells (67.3% versus wild type). These results indicate that heterogeneity of the clinical presentation in SW is caused by the effects of mutation on the substrate-dependent enzymatic activities.

## Discussion

We established a method to easily evaluate enzymatic activity of HSD3B2 to multiple substrates using the reporter assays in cultured cells. This system is highly sensitive and rapidly detects the conversion of substrates, due to a strong response to P4 and A4 from ectopic expression of PR and AR in CV-1 cells, which have a low background activity of C3 group nuclear receptors (29). Using this assay, we defined the effects of various missense mutations on enzymatic activities of human HSD3B2 to both P5 and DHEA.

Human PR was activated by P4 from 10^-9^ M. This concentration is lesser than plasma progesterone concentration in men and women at follicular phase, whereas it elevates to over 10 nM at luteal phase (30). Therefore, this concentration acts as a critical point for the actions of progesterone in menstrual cycle and early pregnancy, despite it continues to increase additional several tens-fold for maintaining the pregnancy until late phase (31–33). In contrast, A4 strongly activated human AR from 10^-8^ M. Because plasma concentrations of A4 is just less than this value in both men and women throughout the life (30), these facts are consistent with the concept that A4 represents as an important intermediate for sex steroids, such as testosterone and estrogens (34, 35). However, it was reported that A4 exceeds this concentration in the deficiency of some steroidogenic genes, such as HSD17B3 (36, 37) and CYP21A2 (38, 39). Although it is possible that A4 acts as an androgen in these patients, the action is insufficient for complete masculinization. This is due to the necessity of 5α-dihydrotestosterone converted from T by 5α-reductase in peripheral tissues for the male sexual differentiation (40, 41). Nevertheless, it is not ruled out that elevated A4 contributes to the partial virilization in CYP21A2-deficient female patients, even though 11-oxygenated androgens, such as 11-ketotestosterone, are likely most important (38, 42), Further detail studies are necessary to reveal the characteristics of this weak androgen in sexual development.

It is interesting that conversion of DHEA into A4 was markedly reduced in all mutants, whereas conversion of P5 into P4 was markedly reduced in three mutants (C72R, S124G and V225D). Although the conversion was also reduced in V299I, the activity was maintained at far more than 50% compared to wild type. This result is consistent with the clinical phenotypes; all the patients have ambiguous genitalia, whereas SW was observed in all the patients except for V299I (25–28). As far as we know, this is the first report revealing the different effects of amino-acid substitution of HSD3B2 on enzymatic activities by the substrates. V299 is the component of the putative membrane spanning domains that are possibly involved in the substrate-specificity (43). Therefore, it is probable that other mutation in these domains show the fluctuation of enzymatic activity dependent on the substrates as V299I. However, enzymatic activities of mutant proteins have been often evaluated using only one substrate in previous studies (44–47) Because the clinical manifestation of HSD3B2 gene mutations is heterogenous between patients, evaluation of enzymatic activities using multiple substrates is necessary for accurate diagnosis.

To resolve the problems of ambiguous genitalia by HSD3B2 deficiency, testosterone replacement therapy have been performed in the male patients, including C72R and V225D patients (27, 28, 48). This treatment attenuates microphallus by promoting the penile growth. In contrast, V299I patient suffered from premature pubarche with rapid axially/public hair and phallic enlargement at 7 years of age, despite he also showed ambiguous genitalia at birth (25). Consistent with the clinical features, plasma testosterone concentration was markedly increased. It was also reported in previous studies that parts of male patients with HSD3B2 mutations spontaneously enter puberty (45, 49–53). Although contribution of peripheral HSD3B1with aid of HSD17B5 is predicted in such patients (44), it is also possible from our results that elevation of testosterone levels is caused by the residual enzymatic activities and up-regulation of adrenal HSD3B2 expression through the elevation of ACTH in CAH (54). In fact, various mutant proteins sustain the residual activities (44). Therefore, it is conceivable again that evaluation of the enzymatic activities of mutant proteins is essential for the accurate diagnosis in this disease.

In summary, we developed a method to evaluate the enzymatic activity of 3β-HSDs to multiple substrates. It provides a useful method for comprehensive analyses of HSD3B2 mutant proteins that cause heterogenous clinical features. In addition, this system could also be useful for evaluating the enzymatic activities of HSD3B1 to develop new drugs. Although the mutations in HSD3B1 have not been linked to any genetic diseases, it was reported that its SNPs are associated with breast and prostate cancers (7–13). Therefore, it could be one of the therapeutic targets for preventing the progression of these diseases. Because the present system can easily examine the effects of added compounds through cell-based reporter assay, it could be applied to identify the compounds to suppress its activity and disease progression.

## Data Availability

The original contributions presented in the study are included in the article/**Supplementary Material**. Further inquiries can be directed to the corresponding author.
