# Supplementary material for: Evaluation of 3β-hydroxysteroid dehydrogenase activity using progesterone and androgen receptors-mediated transactivation
Source: Front Endocrinol (Lausanne). 2024 Oct 2;15:1480722. doi: 10.3389/fendo.2024.1480722 (PMC11479897; doi:10.3389/fendo.2024.1480722)
Supplement: Supplementary file 1 [file DataSheet1.pdf]

Supplementary Table 1. Primers used in mutagenesis of HSD3B2 gene. Small letters indicate the substituted base sequence.

**PCR primers**	**Forward primers**	**Reverse primers**
human HSD3B2-C72R	F- CTGAAAAGAGCCcGCCAGGACGTCT	R- AGACGTCCTGGCgGGCTCTTTTCAG
human HSD3B2-S124G	F- TTCATCTACACCgGTAGCATAGAGG	R- CCTCTATGCTACcGGTGTAGATGAA
human HSD3B2-V225D	F- ACCCAGTCTATGaTGGCAACGTGGC	R- GCCACGTTGCCAtCATAGACTGGGT
human HSD3B2-V299I	F- CTGCTGGAAGTAaTGAGCTTCCTAC	R- GTAGGAAGCTCAtTACTTCCAGCAG

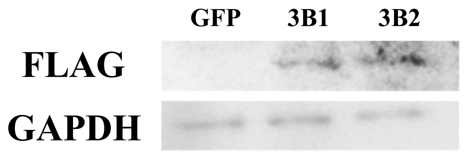

Supplementary Figure 1. Expression of FLAG-tagged human HSD3B1 and HSD3B2 in each gene-transfected HEK293 cells. Western blot analyses were performed with the antibodies against FLAG and GAPDH using lysates derived from GFP-, HSD3B1- or HSD3B2-introduced HEK293 cells.

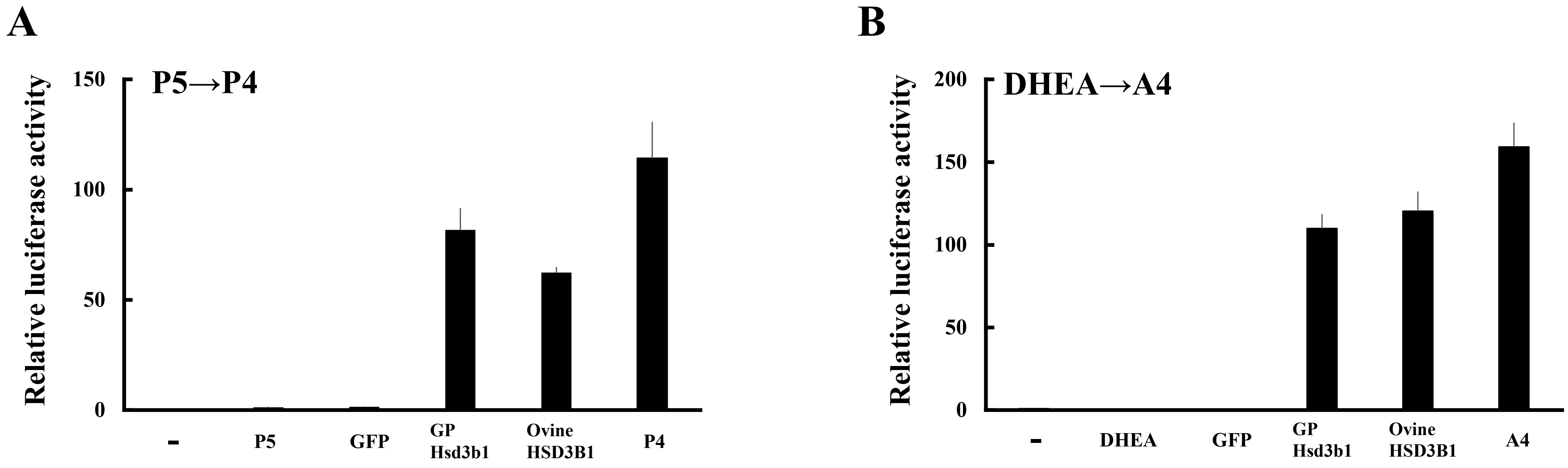

Supplementary Figure 2. Evaluation of the enzymatic activities of guinea pig (GP) Hsd3b1 and ovine HSD3B1 using culture media from each gene-transfected HEK293 cells. Activation of human PR- and AR-mediated transcription by HSD3B1 and HSD3B2 using culture media from each gene-transfected HEK293 cells. CV-1 cells were transfected with PRE-Luc/ human PR-expression vectors (A) and ARE-Luc/ human AR-expression vectors (B). At 24 h post-transfection, cells were incubated for 24 h with vehicle (lane C), P5 (10 nM), P4 (10 nM), DHEA (10 nM),A4 (10 nM), culture medium from GFP or each HSD3B-expressing HEK293 cells collected at 2 h and 3 h after addition of P5 (10 nM) and DHEA (10 nM), respectively. Values of the vehicle were defined as 1. Data represent the mean ± SEM of at least four independent experiments.
